# Performance of the 2010 Classification Criteria for Rheumatoid Arthritis: A Systematic Literature Review and a Meta-Analysis

**DOI:** 10.1371/journal.pone.0056528

**Published:** 2013-02-20

**Authors:** Garifallia Sakellariou, Carlo Alberto Scirè, Antonella Zambon, Roberto Caporali, Carlomaurizio Montecucco

**Affiliations:** 1 Chair and Division of Rheumatology, IRCCS Policlinico San Matteo Foundation, Pavia, Italy; 2 Department of Biostatistics Statistics and Quantitative Methods, University of Milano-Bicocca, Milano, Italy; Universidad Peruana de Ciencias Aplicadas (UPC), Peru

## Abstract

**Objectives:**

To evaluate the performance of the American College of Rheumatology (ACR)/European League Against Rheumatism (EULAR) 2010 classification criteria for rheumatoid arthritis (RA) with a systematic literature review and a meta-analysis.

**Methods:**

PubMed, Embase, Cochrane Library and the abstracts of the ACR and EULAR meetings (2010–2012) were searched for original articles or abstracts with the following inclusion criteria: 1) recent onset arthritis, with at least one swollen joint and no alternative diagnosis; 2) the ACR/EULAR 2010 criteria as index test; 3) the prescription of methotrexate (MTX) or disease modifying antirheumatic drugs (DMARDs) at any time during follow-up as reference standard. Data were pooled using the bivariate model. Three meta-analyses were performed with MTX (primary analysis), DMARDs or their combination (secondary analyses) as reference standard. Heterogeneity was formally tested and explored performing an influence analysis.

**Results:**

The search identified 1,277 references. Six full papers and 4 abstracts met the inclusion criteria. With MTX as reference standard, sensitivity (95% confidence interval, CI) was 0.80 (0.74,0.85), specificity 0.61 (0.56,0.67), positive likelihood ratio (LR) 2.11 (1.92,2.32), negative LR 0.31 (0.25,0.38) and the diagnostic odds ratio (DOR) was 6.74 (5.49,8.28). Using DMARDs as reference standard, sensitivity was 0.73 (0.64,0.80), specificity was 0.74 (0.68,0.79), LR+2.85 (2.53,3.22), LR− 0.35 (0.27,0.45) and DOR 8.03 (6.4,10.09). Using the combination of MTX and DMARDs as reference standard, intermediate results were obtained. The influence analysis detected one potentially influential study. However, its exclusion from the meta-analysis did not have a clinically relevant impact on the results.

**Conclusions:**

The new classification criteria have good sensitivity, lower specificity and an overall moderate diagnostic accuracy. These results confirm that the criteria have classificative and not diagnostic function.

## Introduction

In the last few decades, the recognition of the central role of an early diagnosis and the early administration of disease modifying antirheumatic drugs (DMARDs), particularly methotrexate (MTX), greatly improved the management of rheumatoid arthritis (RA) [1]. The more effective diagnostic and treatment strategies led to a better control of the disease with a deeper suppression of synovitis [2] and the prevention of radiological progression of bone erosions in the joints [3]. All these improvements have led to a great reduction of the risk of permanent disability [4], [5] which is the most significant long-term detrimental consequence of the disease.

For these reasons, the early recognition of RA has become a central issue in clinical practice, although the absence of a single and reliable test to identify the disease does not always allow an immediate diagnosis. The American College of Rheumatology (ACR) proposed in 1987 a set of classification criteria, developed in patients with longstanding disease with the aim to be specific rather than sensitive [6]. These criteria, that were initially meant for the enrolment of patients in clinical trials, but in some cases are used for diagnosis, have shown an unsatisfactory performance in the setting of early arthritis, especially due to a low sensitivity [7]. The inadequate performance of the 1987 criteria led in recent years to the development of prognostic algorithms, meant to discriminate, at the time of symptom onset, patients with higher probability of persistent disease amenable to treatment with DMARDs from those with self-limiting arthritis [8], [9].

In 2010 the ACR and the European League Against Rheumatism (EULAR) jointly developed new classification criteria, aiming to allow earlier patient classification, treatment and inclusion in clinical trials [10]. The new criteria were developed from inceptional cohorts of inflammatory arthritis data [11] which subsequently integrated with expert opinion [12] and finally validated in external early arthritis cohorts. For their development, the reference standard for diagnosis was the prescription of MTX or other DMARDs within the first year of observation. This was considered the best available reference standard, reflecting the risk of chronicity and erosive damage [10]. Since the criteria were developed using a reference standard that can be fulfilled after a follow-up, their value is not only classificative, but also prognostic. The opinion of an expert rheumatologist was not considered an acceptable reference standard because the widespread knowledge of the 1987 classification criteria would have likely lead to a circularity bias.

The 2010 criteria include tender and swollen joint count, acute phase reactants, anti-cyclic citrullinated peptide antibodies (ACPA) or rheumatoid factor (RF), and symptom duration (Table 1). These clinical and laboratory data are combined into a score ranging from 0 to 10. In the validation cohorts, the areas under the Receiving Operator Characteristics (ROC) curve (AUC) for the new criteria ranged from 0.66 to 0.82, and the score that determined an optimal discrimination was between 6 and 7. The cut-off of 6 was afterwards chosen in order to improve sensitivity. However, detailed information on the sensitivity and specificity of the new criteria, along with the corresponding likelihood ratios (LR), has not been reported in the original paper. Moreover, there was a certain variability in accuracy measures across different cohorts [13].

**Table 1:** 2010 ACR/EULAR classification criteria for rheumatoid arthritis.

**A Joint involvement**	
1 large joint	0
2–10 large joints	1
1–3 small joints	2
4–10 small joints	3
>10 joints	5
**B Serology**	
Negative RF and negative ACPA	0
Low-positive RF or low-positive ACPA	2
High-positive RF or high-positive ACPA	3
**C Acute-phase reactants**	
Normal CRP and normal ESR	0
Abnormal CRP or Abnormal ESR	1
**D Duration of symptoms**	
<6 weeks	0
≥6 weeks	1

The criteria are meant to be applied in patients with at least one swollen joint, after the exclusion of other causes of synovitis. Patients with a score ≥6 are classified as having RA. Also subjects with typical bone erosions can be classified as RA regardless of the score. Modified from Aletaha D, et al. RF: rheumatoid factor; ACPA: anti-cyclic citrullinated peptide antibodies; CRP: C-reactive protein; ESR: erythrocyte sedimentation rate.

After their first presentation at the ACR congress in 2009, the new criteria have been tested in a number of external early arthritis cohorts with a wide variability in the overall performance [14]–[17]. These studies are slightly different in terms of population, but the main differences are in the assessment of the reference standard. In fact, many studies considered expert opinion as reference standard [15], as well as disease persistency, appearance of bone erosions or the 1987 classification criteria [17]. Only some of the studies considered the prescription of MTX or other DMARDs as reference standard for diagnosis as indicated in the newly developed criteria.

Because of the increasing knowledge on the 2010 RA classification criteria it seemed timely to summarise the results of the available literature. The present study aims to evaluate the performance of the 2010 ACR/EULAR classification criteria in populations of individuals with early arthritis. For this purpose, we performed a systematic literature review and subsequent meta-analyses in which we considered the 2010 classification criteria a s index test, the prescription of MTX or other DMARDs as reference standard for the classification of RA as done during the development of the criteria.

## Methods

The Preferred Reporting Items for Systematic Reviews and Meta-analyses (PRISMA) guidelines for the reporting of systematic reviews and meta-analysis were followed to conduct this review [18]. A pre-specified protocol, including research question, search strategy, inclusion criteria for the articles and methods for the analysis, was developed before the beginning of the study.

### Data Sources and Search

The search was performed by one of the authors (GS) and a control search by a second author (CAS). We searched Medline (PubMed), Embase and Cochrane databases from November 2009 (when the criteria were presented for the first time) to May 2012. The search strategies are shown in table 2. The search strategy was based on terms related to RA, classification and diagnosis. The references of the included studies were manually screened to search for further papers. The abstracts of the ACR (2010 and 2011) and EULAR congresses (2010, 2011, 2012) were examined to look for additional studies. No language or publication restrictions were applied and studies were not selected based on quality. Filters developed for the identification of diagnostic studies were not utilised since such filters may result in omission of relevant studies [19].

**Table 2:** Search strategy.

**PubMed**	1 “rheumatoid arthritis”
	2 Arthritis, rheumatoid[Mesh]
	3 #1 OR #2
	4 “classification”
	5 “diagnostic criteria”
	6 “ACR EULAR”
	7 #4 OR #5 OR #6
	8 #3 AND #7
**Embase**	1 ‘rheumatoid arthritis’/exp AND [embase]/lim
	2 ‘classification criteria’ AND [embase]/lim
	3 acr AND eular
	4 #2 OR #3
	5 #1 AND #4 AND [embase]/lim AND [1-6-2009]/sd AND [2009–2012]/py
**Cochrane**	#1 MeSH descriptor Arthritis, Rheumatoid, this term only
	#2 rheumatoid arthritis
	#3 (#1 OR #2)
	#4 classification
	#5 diagnostic criteria
	#6 ACR EULAR
	#7 (#4 OR #5 OR #6)
	#8 (#3 AND #7)

Limits: humans, adults, from November 2009.

### Study Selection

Studies should include subjects presenting with recent onset arthritis, with at least one swollen joint and no definite diagnosis that could explain symptoms (that is, the same population in which classification criteria should be applied) [10]. Finally, only data on patients with RA or undifferentiated polyarthritis (UPA) at baseline were included. The 2010 classification criteria were the index test, in the score format; the presence of bone erosions was not included since it was not tested in the data-driven phase of the development. Moreover, a definition of typical erosions had not yet been presented during the timespan that we examined. The prescription of MTX or DMARDs was considered as reference standard. In particular, we included studies that considered MTX (alone) as reference standard or overall DMARDs (including also MTX). Corticosteroids were not included.

Diagnostic cohort studies (prospective and retrospective) and case control studies were eligible for inclusion in the review. The presence of sufficient data to build a 2×2 table of diagnostic performance was required. Two reviewers independently screened titles and abstracts. The full-text of the potentially eligible articles was obtained; inclusion assessment was performed by one reviewer and checked by a second. Disagreements were resolved by consensus. Only the most recent and complete report was included in the case of studies reported in multiple publications or abstracts.

### Data Extraction and Quality Assessment

We extracted data using a standardised form. Results were extracted as 2×2 tables. Studies were assessed for methodological quality by using the modified version of the Quality Assessment of Diagnostic Accuracy Studies (QUADAS) tool proposed by the Cochrane Collaboration [20]. Data extraction and quality assessment were done by one reviewer and checked by a second reviewer.

### Data Synthesis and Analysis

Sensitivity and specificity were calculated for each 2×2 set of data. Heterogeneity among *n* included studies was visually evaluated plotting sensitivity and specificity on a ROC graph, and separately tested by the Chi-square test using *n*−1 degree of freedom [21].

In the presence of heterogeneity and negative correlation between sensitivity and specificity, as commonly seen in diagnostic studies, the bivariate model was used to estimate summary sensitivity and specificity with 95% confidence intervals (CI) and to derive a hierarchical summary receiving operator characteristic (HSROC) curve [22]–[24].Summary positive and negative likelihood ratios (LR+, LR−) and the diagnostic odds ratio (DOR) were derived for each analysis.

Three different analyses were performed. Primary analysis used MTX as reference standard for diagnosis. Secondary analysis used DMARDs as reference standard for diagnosis. Since MTX is a particular type of DMARDs, and in order to increase the number of available studies and hence the precision of the meta-analysis, a third analysis used MTX or DMARDs as reference standard was performed. For this analysis, if data on both MTX and DMARDs were reported separately, data on DMARDs were used.

To evaluate the robustness of our findings, we performed an influence analysis calculating Cook’s distance for each study. This index is a measure of the influence of a study on the model parameters and can be used to check for particularly influential study. Studies with Cook’s distance ≥1 were considered potentially influent. A further meta-analysis was then performed after excluding these potentially influential studies.

Publication bias was indirectly evaluating the symmetry on the funnel plot of logDOR. Forest plots were used to represent sensitivity and specificity of the primary studies.

Finally to verify the presence of publication bias, a funnel plot was implemented.

Review Manager (RevMan) version 5 (The Nordic Cochrane Centre, The Cochrane Collaboration, Copenhagen) was used to build the forest plot showing sensitivity and specificity of single studies and the risk of bias tables. Stata, version 11, (StataCorp, College Station, Texas) was used to perform all analyses. In particular the generalized linear mixed model approach to bivariate meta-analysis of sensitivity and specificity was obtained using the ‘metandi’ command of Stata.

## Results

**Figure 1:**
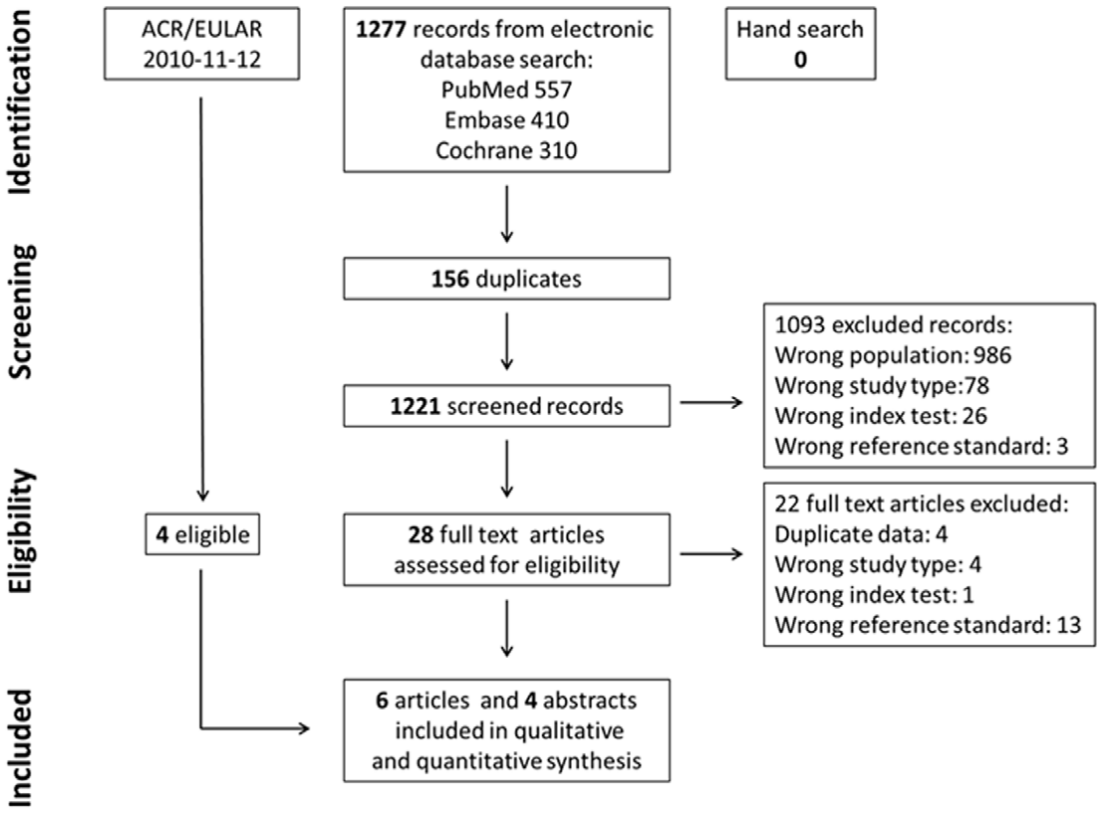
PRISMA flow-chart describing the selection process in the systematic literature review.

**Figure 2:**
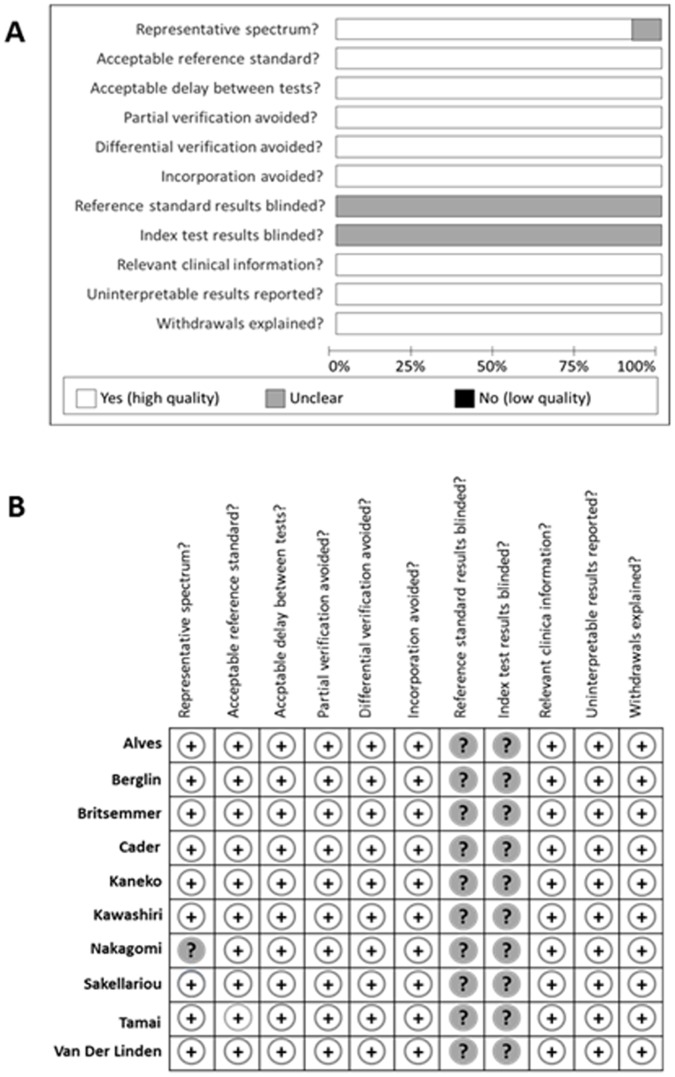
Sensitivity and specificity of the included studies. A) methotrexate as reference standard **B)** disease modifying antirheumatic drugs as reference standard **C)** methotrexate+ disease modifying antirheumatic drugs as reference standard.

The search strategy identified 1277 studies. Figure 1 reports the flow-chart of the process of study selection. 6 articles were included after the evaluation of all titles and abstracts [14], [16], [25]–[28]. Moreover, 4 abstracts from the ACR and EULAR congresses were eligible for inclusion [29]–[32]. In total, 4 papers used MTX as unique reference standard, 3 used DMARDs as unique reference standard, while 3 studies reported separate data on both MTX and DMARDs. Two studies [15], [33] were excluded since they used a composite reference standard based on concomitant expert opinion and DMARDs and expert opinion was not considered an acceptable reference standard for this review. Table 3 and Figure 2 summarise the main features of the included studies. Figure 2 shows the point estimates with 95% CIs of sensitivity and specificity for the included studies. Sensitivity ranged from 0.68 to 0.88 when MTX was considered as reference standard, and from 0.59 to 0.87 when DMARDs were used. The specificity ranged from 0.50 to 0.72 with MTX as reference standard, and from 0.64 to 0.88 with DMARDs.

**Table 3:** Characteristics of the included studies.

Study	Clinical setting	Population	Calendar year	Index test	Reference standard	Study design	N	Follow-up duration	RF/ACPA (%)	Symptoms duration	Joint count
Alves	Early arthritis	≥1 swollen joint for less than 1 year; ≥16 years old, no history of trauma or exertion. MTX: 64/231	2004–2008	ACR/EULAR 2010 classification criteria	MTX	Retrospective cohort	231	12 months	26/19	3.47 months	44
Berglin	Early arthritis	≥1 swollen joint; no alternative diagnosis; ≤1 year symptom duration; ≥1 year of follow-up. MTX: 230/313	n.a.	ACR/EULAR 2010 classification criteria	MTX	Retrospective cohort	313	12 months	60/64	4 months	n.a.
Britsemmer	Early arthritis	>18 years old; ≥2 swollenjoints; symptom duration of<2 years; no prior DMARDs.MTX: 354/455	2000–n.a.	ACR/EULAR 2010 classification criteria	MTX	Retrospective cohort	455	12 months	42.6/52	5.6 months	n.a.
Cader	Early arthritis	≥1 swollen joint;; ≤3 months of symptom duration; ≥18 months follow-up. MTX: 74/205; DMARDs: 102/205	n.a.	ACR/EULAR 2010 classification criteria	MTX DMARDs	Retrospective cohort	205	18 months	56/59	1.37 months	66
Kanenko	Early arthritis	joint symptoms (arthralgia, joint swelling and morning stiffness); no previous treatment; no diagnosis DMARDs: 54/82	2009–2010	ACR/EULAR 2010 classification criteria	DMARDs	Retrospective cohort	82	0–3 months	66/61	4 months	n.a.
Kawashiri	Early arthritis	suspicion of RA; symptom duration <1 year; no alternative diagnosis; no previous DMARDs. DMARDs: 37/69	2010–2011	ACR/EULAR 2010 classification criteria	DMARDs	Prospective cohort	69	3 months	49/36	4 months	n.a.
Nakagomi	Early arthritis	musculoskeletal symptoms <3 years; no alternative diagnosis. MTX:83/117	n.a.	ACR/EULAR 2010 classification criteria	MTX	Prospective cohort	117	12 months	n.a./n.a.	n.a.	28
Sakellariou	Early arthritis	patients with RA or UPA. MTX: 202/26; DMARDs: 211/266	2005–2010	ACR/EULAR 2010 classification criteria	MTX DMARDs	Retrospective cohort	266	12 months	33/n.a.	3.07 months	44
Tamai	Early arthritis	early arthritis; no alternative diagnosis. DMARDs: 97/138	n.a.	ACR/EULAR 2010 classification criteria	DMARDs	Prospective cohort	138	12 months	n.a.	3 months	n.a.
Van der Linden	Early arthritis	arthritis confirmed by physical examination; <2 years symptom duration. MTX: 445/2258; DMARDs: 1066/2258	1993–2009	ACR/EULAR 2010 classification criteria	MTX DMARDs	Retrospective cohort	2258	12 months	30/29	5.7 months	66

N: number of patients included; n.a.: not available; RF: rheumatoid factor; ACPA: anti-ciclic citrullinated peptide antibodies; MTX: methotrexate; DMARDS: disease modifying antirheumatic drugs; RA: rheumatoid arthritis; UPA: undifferentiated polyarthritis.

**Figure 3:**
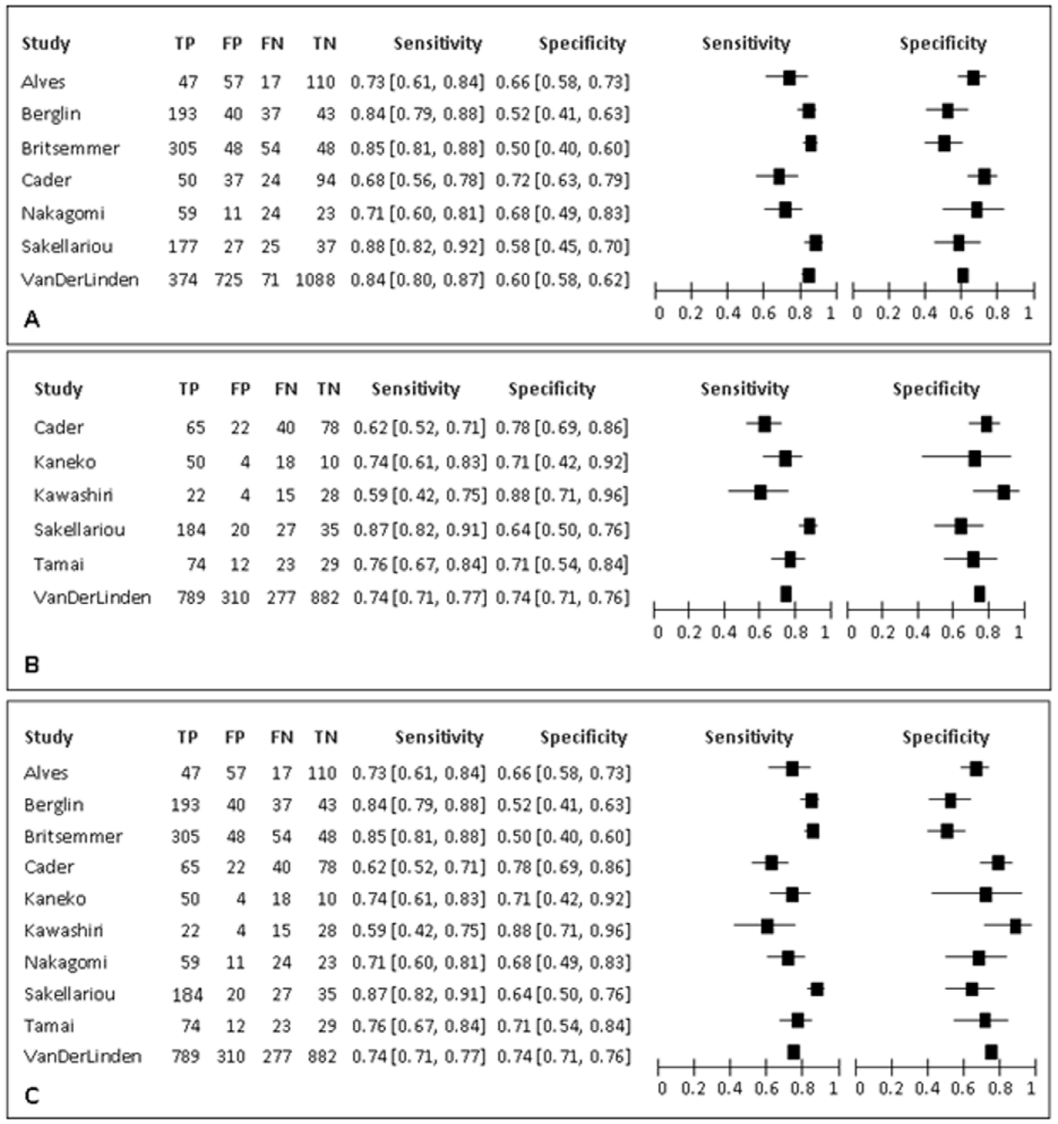
Assessment of the risk of bias in the included studies. 3A) Methodological quality graph: authors’ judgment about each methodological quality item presented as percentages across all included studies. 3B) Methodological quality summary: authors’ judgment about each methodological quality item for each included study. +: low risk of bias; ?: unclear risk of bias; -: high risk of bias.

The evaluation of the methodological quality of the included studies is shown in Figure 3. All studies had a low risk of bias for most of the items except for blinding that was not explicitly mentioned in any of the studies. For this reason, the blinding of the results of the reference standard and the index test was considered unclear. All the included studies had an overall low risk of bias; for this reason, additional analyses, excluding those for low-quality studies, were not performed, since the results of the meta-analysis would not be affected by a low methodological quality.

Both sensitivity and specificity showed significant heterogeneity, with a Chi-square for differences across studies of 29.01 (p<0.0001) and 17.09 (p = 0.009) respectively.

Table 4 summarises the results of all the meta-analyses. Using MTX as reference standard, pooled sensitivity (95% CI) was 0.80 (0.74, 0.85) and pooled specificity (95% CI) was 0.61 (0.56, 0.67), a LR+ of 2.11 (1.92, 2.32) and a LR− of 0.31 (0.25, 0.38).

**Table 4:** Results of meta-analyses.

Reference standard	Studies (n)	Sensitivity (95% CI)	Specificity (95% CI)	LR+(95% CI)	LR– (95% CI)	DOR (95% CI)
***MTX***	7	0.80 (0.74,0.85)	0.61 (0.56,0.67)	2.11 (1.92,2.32)	0.31 (0.25,0.38)	6.74 (5.49,8.28)
***DMARDs***	6	0.73 (0.64,0.80)	0.74 (0.68,0.79)	2.82 (2.53,3.22)	0.35 (0.27,0.45)	8.03 (6.40,10.09)
***MTX+DMARDs***	10	0.76 (0.71,0.81)	0.69 (0.61,0.75)	2.48 (2.08,2.95)	0.33 (0.29,0.38)	7.38 (6.33,8.62)

95% CI: 95% confidence interval; LR+: positive likelihood ratio; LR−: negative likelihood ratio; DOR: diagnostic odds ratio; MTX: methotrexate; DMARDs: disease modifying antirheumatic drugs.

Using DMARDs as reference standard, sensitivity was 0.73 (0.64,0.80), specificity was 0.74 (0.68,0.79), LR+ was 2.85 (2.53,3.22) and LR− was 0.35 (0.27,0.45).

**Figure 4:**
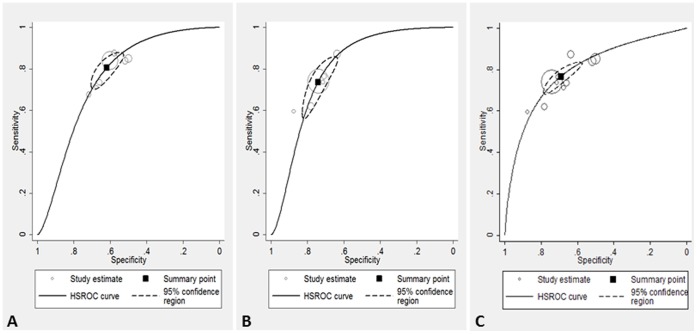
Results of the meta-analyses. A) methotrexate as reference standard; 7 studies included, 3845 participants. **B)** disease modifying antirheumatic drugs s as reference standard; 6 studies included, 3018 participants. **C)** methotrexate+disease modifying antirheumatic drugs as reference standard; 10 studies included, 4134 participants. The black square indicates the point estimate of sensitivity and specificity for the meta-analysis, the dotted line indicates the 95% confidence interval. The continuous line is the hierarchical summary receiving operator characteristics curve. The dots represent the primary studies.

The third analysis, combining MTX and DMARDs, led to intermediate results (Figure 4).

**Figure 5:**
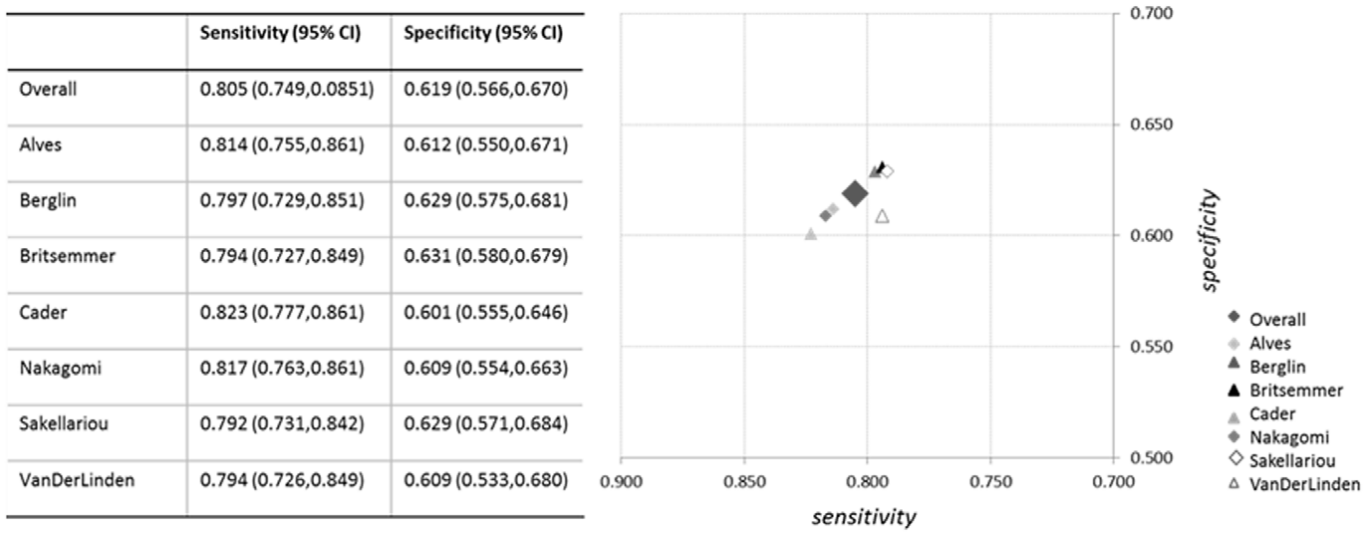
Results of meta-analyses excluding single studies. Overall meta-analysis, based on the primary analysis using methotrexate as reference standard, and meta-analysis excluding a single study are reported. The table and the legend refer to the study that has been excluded.

The influence analysis using the Cook’s distance identified Van Der Linden’s study as a potentially influential [27]. After removing this study the pooled sensitivity changed from 0.80 (0.74, 0.85) to 0.79 (0.72, 0.84), and specificity from 0.61 (0.56, 0.67) to 0.60 (0.53, 0.68). The influence of each single study is reported in Figure 5.

**Figure 6:**
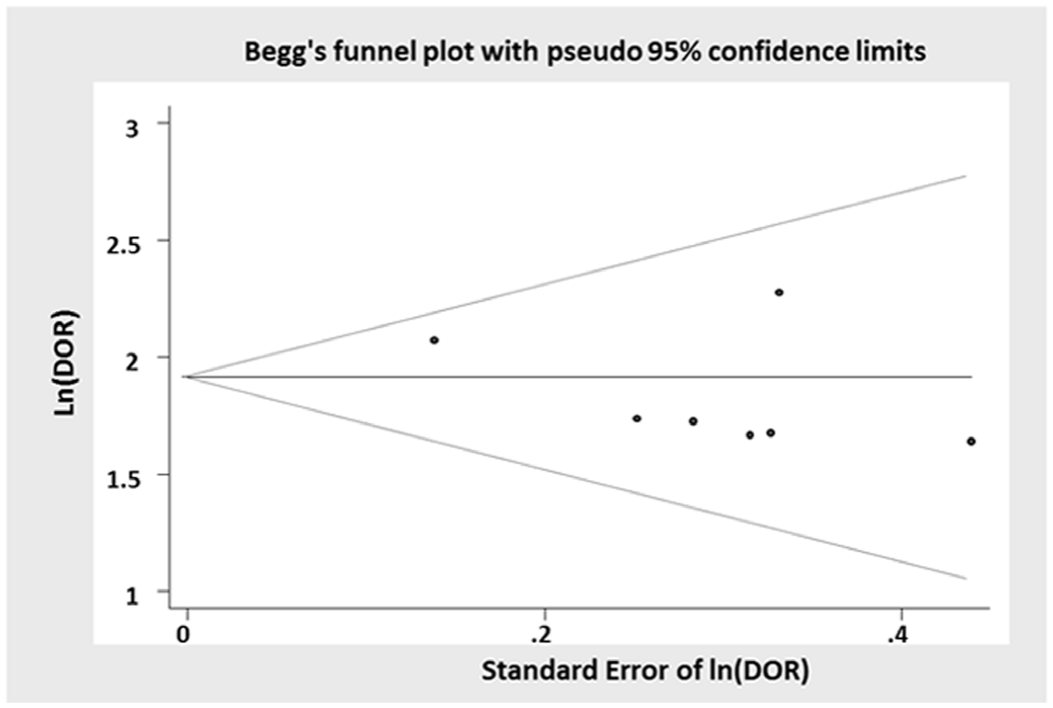
Funnel plot. The distribution of the studies in the funnel plot does not suggest the presence of publication bias. In fact, studies are distributed by each side of the plot, with moreover a lower number of studies with positive results. In the case of publication bias, the opposite situation would have been expected.

Funnel plot did not show any systematic asymmetry suggesting the absence of publication bias (Figure 6).

## Discussion

Since the new classification criteria for RA were first presented, several studies have provided a wide range of results by evaluating their performance in the setting of early arthritis. These studies were different in terms of population (mainly due to the recruitment criteria and calendar year into the existing cohorts) and reference standard. Moreover, treatment patterns differed significantly among them. This variability did not allow the drawing of consistent conclusions from a single study. Therefore it seemed appropriate to review the available literature on this topic.

We adopted a search strategy meant to be sensitive rather than specific; the limited time period under exam allowed this approach. We did not use pre-specified filters for diagnostic accuracy studies [19]. Since the search was designed to be as broad as possible, it is unlikely that relevant studies were missed. Even in this way, the number of studies that were eligible for the inclusion was limited. This is the main limitation of this review, since it did not allow to investigate the causes of heterogeneity in detail and, for the same reason, subgroup analyses were not feasible.

The majority of the validation studies did not use the same reference standard highlighted in the development of the criteria and many of them used expert opinion or the 1987 ACR criteria. In particular, 13 studies that were selected for detailed review have afterwards been excluded because of a wrong reference standard. These studies were excluded because of the possibility of the introduction of considerable circularity bias. Same reference standard used to develop the new criteria were adopted, that is the prescription of MTX or other DMARDs [10]. The results obtained showed an overall moderate performance of the criteria with acceptable values of sensitivity through all reference standards. One the other hand, specificity was lower in the analyses based on MTX, and MTX with DMARDs. The analysis based on overall DMARDs as reference standard showed a better specificity of the 2010 classification criteria. In fact, MTX might not have been given to all patients that would require it due to contraindications or national differences in prescriptions. On the other hand sensitivity does not increase accordingly because a twofold higher increase of subjects treated with DMARDs not fulfilling the 2010 criteria is observed.

The values of LR+ were around 2.5, far from the values of LR+ >10 that ideally identify a test having the power to detect the disease. However, LR− were around 0.3, which is closer to the optimal values of LR− <0.1 [34]. This indicates the patients not fulfilling the 2010 classification criteria have high probability of not having RA (and will not develop it later, if we consider that the criteria identify patients that might fulfil an outcome after a follow-up), while patients fulfilling them will not certainly develop RA. In contrast with this consideration, the DORs suggest a good performance, though this is probably driven by low values of LR−.

The need to perform differential diagnosis before the application of the criteria might have determined the underestimation of their accuracy even if the majority of studies excluded patients that were afterwards diagnosed with disease other than RA or UPA. Differential diagnosis is not always feasible in the early stages; this might have increased the number of false positives resulting in the reduction of specificity.

In the original presentation paper, detailed data on sensitivity, specificity and performance were not reported. The values of the areas under the ROC curve (AUC) reported in the validation cohorts ranged from 0.66 to 0.82. The best performance was achieved in the Norwegian cohort of early arthritis, while in the remaining two the AUCs were <0.7; this indicates a test with moderate performance [10]. The differences between the populations could be due to differences in recruitment and treatment. In the external validation cohorts, only the proportion of patients correctly classified was reported, while overall estimates of accuracy were not presented. Since not all information on the performance of the 2010 criteria is provided in the original paper, results could not be directly compared our with data from the inception of the criteria.

As expected, significant heterogeneity was found among diagnostic studies. This finding did not permit the separate combination of sensitivity and specificity. For this reason we adopted a bivariate model that allows for the negative correlation between sensitivity and specificity. Given the low number of studies, investigating heterogeneity by stratified meta-analysis or meta-regression was not feasible. For this reason we evaluated the robustness of our results by performing an influence analysis [21]. However, though one study was demonstrated to be potentially influential, a subsequent meta-analysis after the exclusion of the influential study led to comparable results.

All the included studies had an overall good methodological quality. The items dealing with blinding were not explicitly addressed by the papers, therefore the risk of bias related to these points was considered unclear for all studies. Despite this, given to the retrospective design and the unavailability of the criteria at the time of the clinical assessment and therapeutic decision, this did not clearly biased the results. The last two items (on the report of uninterpretable results and withdrawals), even though not directly reported, were considered satisfactory, since, as suggested by the Cochrane collaboration, they probably had no influence on the results [20]. Since the methodological quality of all the included studies was good, the results of the meta-analysis have likely not been affected by the methodological quality of the primary studies.

A meta-analysis on the diagnostic performance of the 1987 ACR classification criteria demonstrated an overall sensitivity and specificity of 0.77 in the subgroup of patients with early arthritis and a better performance among cases of established arthritis [7]. These results are not substantially different from those of our meta-analyses: the new criteria, specifically designed for early arthritis, do not perform better than the old ones in the same setting. However, it has to be kept in mind that the 1987 criteria were developed based on expert opinion while the 2010 criteria used a more practical reference standard, such as the use of specific treatment regimes. Therefore, the 2010 criteria could be more useful in a clinical setting because they classify patients based on a relevant prognostic aspect of the disease with an impact on clinical management. Nevertheless, the function of the criteria is classification, and not diagnosis; based on current knowledge the 2010 criteria should not be used as a guide to start specific treatments for RA.

The external validation studies in the meta-analysis on 1987 criteria had a cross-sectional case-control design that overestimates the accuracy while the cohort study design (such as in the development of the criteria) tends to underestimate accuracy [35], and this might have influenced our results as well.

One of the merits of the 2010 classification criteria is the fact that it is not necessary to reach six weeks of symptom duration to classify a patient as having RA. Only one study [14] applied the new criteria in a population of very early arthritis, but their performance did not seem substantially different that in the remaining early arthritis population.

The creation of an optimal tool for the classification of rheumatic disease, with RA in particular, has always been challenging. This is mainly due to the absence of gold standards for diagnosis. The decision to prescribe either DMARDs or MTX is dependent from expert opinion and reflects a judgement on the severity of arthritis, for this reason their performance does not seem to be superior to the old ones. However developing the criteria avoiding to use expert opinion as reference standard gives an opportunity to innovate clinical practice, and we could expect that the presentation of the new classification criteria might significantly modify the approach to RA. In fact, the results of the present meta-analysis evaluate the 2010 criteria in the setting of the current management of RA by rheumatologists, but the final judgement on the criteria will probably be drawn after prospective studies, evaluating their long-term impact on disease outcomes.
